# Ontogeny of the maxilla in Neanderthals and their ancestors

**DOI:** 10.1038/ncomms9996

**Published:** 2015-12-07

**Authors:** Rodrigo S. Lacruz, Timothy G. Bromage, Paul O'Higgins, Juan-Luis Arsuaga, Chris Stringer, Ricardo Miguel Godinho, Johanna Warshaw, Ignacio Martínez, Ana Gracia-Tellez, José María Bermúdez de Castro, Eudald Carbonell

**Affiliations:** 1 Department of Basic Science and Craniofacial Biology, New York University College of Dentistry; and NYCEP, New York City, New York, USA; 2 Department of Biomaterials and Biomimetics, New York University College of Dentistry, New York City, New York, USA; 3 Centre for Anatomical and Human Sciences, Department of Archaeology and Hull York Medical School, University of York, York, UK; 4 Universidad Complutense de Madrid-Instituto Carlos III (UCM-ISCIII), Centro de Investigacion de la Evolucion y Comportamiento Humanos, Madrid, Spain; 5 Department of Earth Sciences, Natural History Museum, London, UK; 6 Department de Ciencias de la Vida, Universidad de Alcalá, Alcalá de Henares, Spain; 7 Department Geología, Geografía y Medio Ambiente, Fac. de Biología, Ciencias Ambientales y Química, Universidad de Alcalá, Madrid, Spain; 8 Centro Nacional de Investigacion sobre la Evolucion Humana, Burgos, Spain; 9 Institut Català de Paleoecologia Humana i Evolució Social, Tarragona, Spain

## Abstract

Neanderthals had large and projecting (prognathic) faces similar to those of their putative ancestors from Sima de los Huesos (SH) and different from the retracted modern human face. When such differences arose during development and the morphogenetic modifications involved are unknown. We show that maxillary growth remodelling (bone formation and resorption) of the Devil's Tower (Gibraltar 2) and La Quina 18 Neanderthals and four SH hominins, all sub-adults, show extensive bone deposition, whereas in modern humans extensive osteoclastic bone resorption is found in the same regions. This morphogenetic difference is evident by ∼5 years of age. Modern human faces are distinct from those of the Neanderthal and SH fossils in part because their postnatal growth processes differ markedly. The growth remodelling identified in these fossil hominins is shared with *Australopithecu*s and early *Homo* but not with modern humans suggesting that the modern human face is developmentally derived.

## Abstract (web summary)

Unlike modern humans, Neanderthals had large and projecting faces. Here, the authors show that the maxilla of modern humans is distinct from those of the Neanderthal and Middle Pleistocene hominins from Sima de los Huesos because their growth processes differ markedly during the postnatal period.

## Graphical abstract

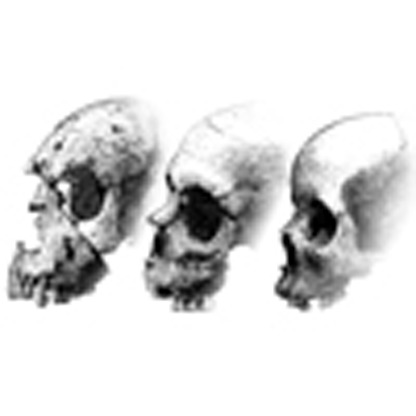

Bone growth remodelling is a key mechanism mediating the development of the facial skeleton^1^,^2^,^3^. It arises from the interplay of osteoblasts which deposit bone, and osteoclasts, which resorb mineralized matrix. This dynamic growth of the facial skeleton in individual species is thus reflected in the main activity present (deposition versus resorption) and the distribution of these growth fields during development. In recent and fossilized facial skeletons, it is possible to map the distributions of these cellular activities through microscopic analysis of bone surfaces^2^,^3^,^4^,^5^,^6^. The developing modern human face mostly presents bone deposition over the upper parts (nasal bones, frontal and zygomatic processes of the zygomatic and nasal processes of the maxillae), whereas the middle and lower face, mainly the anterior maxilla, are commonly dominated by bone resorption starting about a year after birth, which is maintained until adulthood^1^,^7^. This resorption in the human face contributes to the development of key facial characteristics such as maxillary retraction and the development of the canine fossa^1^,^8^,^9^. It is associated with the most antero-inferior point on the face (prosthion) being less anteriorly placed in modern humans than in ‘archaic' *Homo* species including Neanderthals and the SH hominins.

Facial growth remodelling is known for early *Australopithecus* taxa. *Au. afarensis* and *Au. africanus* faces, which share bone deposition throughout the anterior face as the only remodelling activity^2^,^4^,^5^ during ontogeny. Such extensive bone deposition has been implicated in the development of their characteristic facial prognathism^4^. The *Australopithecus* remodelling pattern conforms to the ape pattern^2^,^5^. Early African *Homo* (*H. habilis* and *H. erectus/ergaster*) also display similar growth dynamics to those found in *Australopithecu*s^2^,^3^. However, despite the importance of understanding ontogenetic processes for interpreting differences between *H. sapiens* and other more recent fossils such as Neanderthals^10^ and their ancestors, virtually nothing is known about facial growth remodelling of Middle Pleistocene hominins or the Neanderthals. Such data are potentially important in understanding the timing, extent and mode of possible differences in growth between archaic and modern humans.

Differences in skull form among hominoids have been shown to arise prenatally and to be variably accentuated throughout postnatal life by divergences and differences in magnitudes among subsequent ontogenetic shape trajectories^11^,^12^. With regard to Neanderthals and modern humans, most studies have used 3-D landmark analysis to represent ontogenetic growth^8^,^10^,^13^,^14^. These allow vectors of ontogenetic changes in size and shape to be compared, but differences in vectors do not necessarily relate to differences in bone growth remodelling. Indeed it is possible that similar vectors could be achieved through quite different underlying bone growth activities and rates. Morphometric studies report the consequence of underlying growth processes rather than inform what these processes are. Such studies concluded that the postnatal trajectories of ontogenetic change in face shape do not differ among Neanderthal and modern humans skulls, but that there are differences in the rate of shape change^10^,^15^. These findings are difficult to reconcile with the known differences among geographic groups of modern humans in cranial ontogenetic shape trajectories^16^ in that if modern humans differ among geographic groups, then at least some of these must also differ from Neanderthals. Another study found evidence that postnatal ontogenetic divergence exists between Neanderthal and modern human faces^13^ and this finding has been supported by a further study that compared modern human and Neanderthal mandibular ontogenetic trajectories^14^. Thus, while the initial ontogenetic studies suggested no difference in craniofacial growth vectors of Neanderthals and modern humans^10^,^15^, more recent works have indicated that these two groups differ and that significant differences are also found among living groups of modern humans^11^,^13^,^14^,^16^.

Irrespective of the morphometric studies, it is beyond doubt that differences in size and shape exist among adults of these two groups. Given that the dynamics associated with bone growth remodelling are important in determining the form of the adult face, a finding of a different pattern of remodelling activity between modern humans and the fossil taxa examined in the present study, the SH hominins and Neanderthals, would falsify the hypothesis that these share a common mechanism of postnatal ontogenetic growth. However, these data have been wanting. Here we present, for the first time, a study of facial growth remodelling in two Neanderthal specimens and in four Middle Pleistocene faces from the extensive fossil collection of the Sima de los Huesos (SH) site. We focus our work only on maxillary morphogenesis thus differing from the ontogenetic analysis of the Neanderthal skull previously reported^10^,^15^. Findings from this analysis are interpreted within developmental and evolutionary contexts.

The Devil's Tower (Gibraltar 2) Neanderthal whose death was estimated at ∼4.6 years of age by analysis of enamel microanatomical features^17^, and the slightly older individual of La Quina 18 estimated to be 6–9 years using human dental standards^13^,^18^, were the only juvenile Neanderthals available to us with sufficiently preserved details of the facial bone surface to map the remodelling activity state (see the Methods section). Other specimens included were the Pech de l'Azé child and the adult La Ferrassie 1, but neither preserved the necessary details of the facial bone surfaces. The SH sample includes Cranium 6, Cranium 9, Cranium 16 and the isolated partial face AT-1100. These specimens had an M^2^ either newly erupted (Cranium 9) or showing more advanced wear (see the Methods section), but none had the M^3^ erupted^19^,^20^,^21^. Using recent human dental standards, the age range of the SH sample represents the equivalent of 12–18 years thus allowing us to analyze ontogeny during this period in the SH hominins. Phylogenetically and genetically, the SH palaeodeme is considered to belong to the Neanderthal clade^19^,^20^,^21^,^22^,^23^,^24^. Our results show that Neanderthals and SH hominins show extensive bone deposition over the maxilla, a process associated with midfacial prognathism. This is different from the resorption that dominates over the retracted human maxilla. Thus, the faces of modern humans are distinct from those of the Neanderthal and SH fossils in that the growth processes, as evidenced by anterior maxillary remodelling activity state, differ markedly during the postnatal period.

## Results

### Identification of growth processes

**Figure 1:**
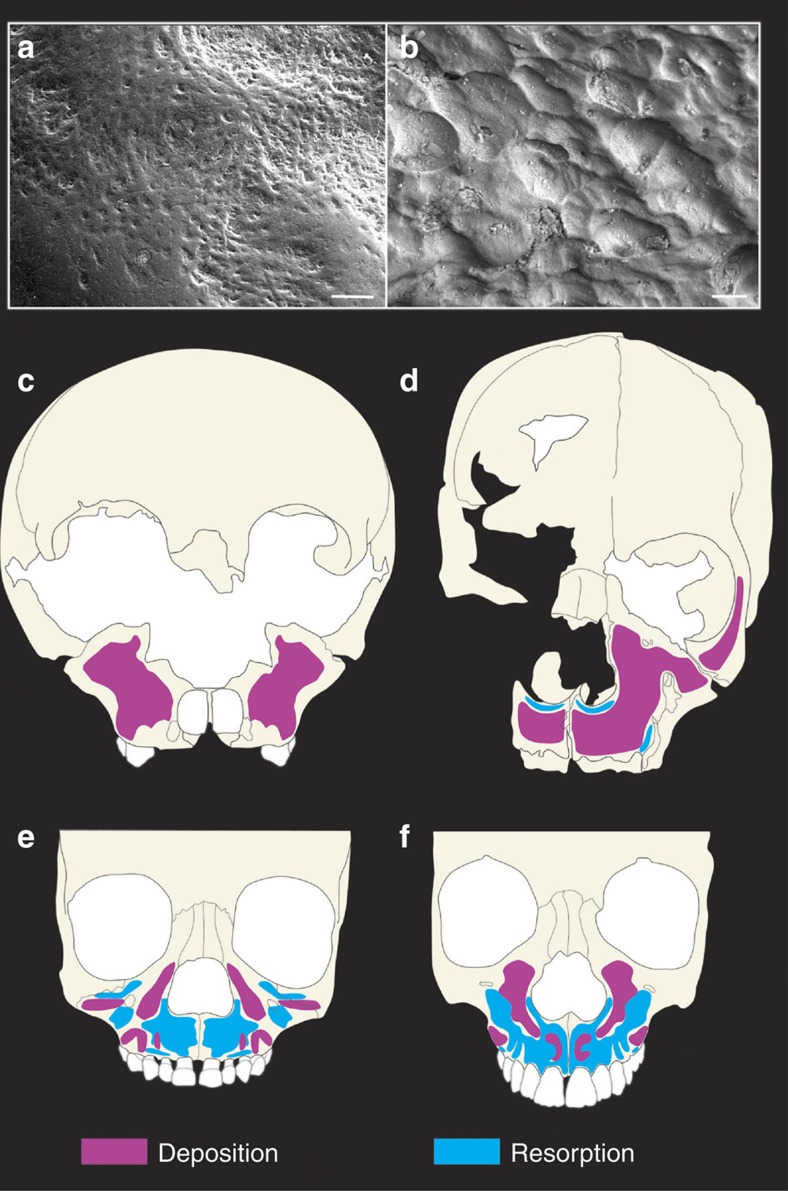
Identification of bone deposition/resorption and facial growth maps. (**a**,**b**) Scanning electron microscope images showing details of bone microanatomy. Scale bars, 100 μm. (**a**) Bone-forming surfaces are relatively smooth, presenting collagen deposits by osteoblast cells. Image taken on the maxillary bone of the Devil's Tower Neanderthal. (**b**) Resorption is identified as irregular surfaces carved by osteoclasts on the bone surface as they dissolve and remove bone matrix. Image taken from the maxillary bone of the SH hominin Cranium 16. (**c**) Facial morphogenetic map of the Neanderthals based on the Devil's Tower child showing only bone deposition over the maxilla. (**d**) Facial morphogenetic map of the SH hominins based on Cranium 9 showing bone deposition over the maxilla with some resorption localized to the entrance to the nasal cavity and lateral maxilla. Remaining SH specimens showed similar facial maps. (**e**,**f**) Facial morphogenetic maps of human sub-adults showing resorption as the dominating feature over the maxilla. These specimens are of similar age to the Devil's Tower child (**e**) and SH Cranium 9 (**f**), respectively.

Specimens studied are shown in Table 1. Identification of the different activity states (bone deposition or bone resorption) on the facial bones of the fossil samples was carried out using scanning electron and portable confocal microscopy (growth remodelling can be investigated using several microscopy techniques, all of which are labour intensive. Most commonly, the scanning electron microscope (SEM) has been used to image remodelling signatures from high-resolution replicas of a specimen, but the environmental SEM can also be used directly on a bone surface if the specimen's size and state of preservation permits. More recently, portable confocal microscopy has enabled imaging surface and subsurface details of bone microanatomy (that is, osteocyte orientation) linked to forming or resorbing activities (see refs 2 and 3). All three techniques were used in this study. Fig. 1, Supplementary Figs 1–3; see also the Methods section). Remodelling fields are binary features presenting forming or resorbing surfaces, and the extent of these activities can be mapped in order to evaluate the contribution of each to facial growth. Thus data interpretation is based on a discernible pattern of bone growth determined by cellular processes which can be defined qualitatively (deposition versus resorption). No statistical test of difference is feasible given the small fossil sample sizes but the fact that the fossils and modern humans show no overlap mitigates the need for such a test. A forming or depository surface differs from a resorptive surface in characteristic ways related to the function of the cell type overlying that bone surface during life. If osteoblasts line the bone surface, matrix is deposited forming a relatively organized tissue, whereas osteoclastic resorptive remodelling forms irregularly spaced and sized lacunae. If the bone surface does not preserve sufficient detail to ascertain the dominant feature owing to damage or fossilization, such surfaces are left unmapped. Fortunately, most of the anterior facial bone surfaces of the specimens studied preserved sufficient microanatomy (in some cases preservation was extraordinary) to allow us to reconstruct the facial bone growth activity state in great detail.

### Neanderthal and SH hominins maxillary remodelling

Our results show that in the Devil's Tower and La Quina 18 Neanderthals (Fig. 1c and Supplementary Fig. 4A, respectively), bone deposition is the only activity state present over the anteriorly facing bone surfaces in the maxilla, including the naso-alveolar clivus, infraorbital and anterior zygomatic. As no resorption could be identified in the preserved areas of these specimens, bone deposition was a key mechanism consistent with the anteriorly directed growth of the maxilla. This extensive depository activity in these Neanderthal fossils differs from the common pattern found in similarly aged *H. sapiens* where resorption is the dominant feature associated with a retracted facial profile^1^,^7^ (Fig. 1e,f). In more than 70 facial skeletons of sub-adult modern humans examined to date^4^,^5^,^7^,^25^, none show the pattern and distribution of activity we have observed in Neanderthals. This extensive depository remodelling activity likely underlies some of the observed differences in early facial ontogeny, and thus also in the adult form, between Neanderthals and modern humans^10^,^14^. Thus the Neanderthal face differs from *H. sapiens* largely because its facial morphogenesis is characterized by different bone growth activity states. The same differences in morphogenesis relative to *H. sapiens* are evident in the SH hominins. In this sample, bone deposition was also the main activity state in the faces of all four SH sub-adults (Fig. 1d and Supplementary Fig. 4). The naso-alveolar clivus, the lateral walls of the nasal opening and the zygomaticomaxillary region (infraorbital plate), as well as the lateral portion of the orbital rim (frontal process of the zygomatic bone) were all depository surfaces. These data highlight important growth similarities shared among the fossil groups studied here but this extensive deposition differs markedly from the mixed depository/resorptive state of the maxillary surface characteristic of the modern human face. Our results also indicate that Neanderthals and modern humans differ in remodelling activity by the time they are ∼5 years of age, this being the approximate dental age of the youngest Neanderthal from which we have been able to recover data on growth remodelling activity state. Differences between the SH hominins and modern humans can only be identified from ∼12 years of age, the estimated age of the youngest specimen in the SH hypodigm found to date.

## Discussion

Knowledge of the biological processes involved in facial ontogeny is key to characterizing and understanding the developmental basis of facial size and shape differences among adults. Bone growth remodelling is one factor that mediates the vectors of growth direction as the facial bones of young individuals increase in size and become displaced in an organized manner to form the adult face. Thus mapping remodelling fields provides an important avenue to assess whether the faces of Neanderthals and humans shared a common ontogenetic trajectory. We have identified growth remodelling patterns in the face of the Devil's Tower and La Quina 18 Neanderthals and in four hominins from SH, a group putatively ancestral to the Neanderthals. As expected, given the constellation of features shared between Neanderthals and the SH hominins, we found that both fossil groups share a common pattern of maxillary bone growth remodelling characterized by extensive bone deposition (Fig. 1c,d). This is in stark contrast to the growth remodelling activity states found over the modern human anterior maxillae, which are mainly resorptive. This resorption is associated with a retracted face in modern humans that is largely positioned under the anterior cranial base^8^. The faces of *H. sapiens* reported to date (*n*=73) ranging from 10 months of postnatal age^7^ to 17 years of age^25^, show resorptive remodelling either extensively distributed or more localized^2^,^5^,^7^,^25^,^26^. In the two Neanderthals analysed here we did not find any evidence of resorption. Thus, despite variation among modern human individuals of varying ages in the extent and duration of remodelling, these Neanderthal specimens show clearly different growth characteristics. A previous analysis of fragmentary mandibular remains of Neanderthals reported a growth remodelling pattern different from modern humans^27^. However, this study had an important limitation with respect to the interpretation of ontogenetic differences in that the sample consisted of adult individuals and mandibular fragments that could not be confidently ascribed to non-adults^27^. This is significant because recent data suggest that remodelling activity state and extent over the anterior maxilla differs between developing and adult individuals of *H. sapiens*^25^.

Our results indicate that even in the youngest Neanderthal examined (∼age 5), different growth remodelling activities to those found in modern humans are apparent. Hence the morphogenetic underpinnings of the Neanderthal facial phenotype already differ from *H. sapiens* by that time, indicating differences in ontogeny. This is consistent with the observation that differences in facial form already exist among the youngest known Neanderthals and modern humans^10^,^13^,^14^,^15^. Differences between the SH hominins and modern humans can only be identified from ∼12 years of age given the lack of younger specimens from the SH hypodigm.

**Figure 2:**
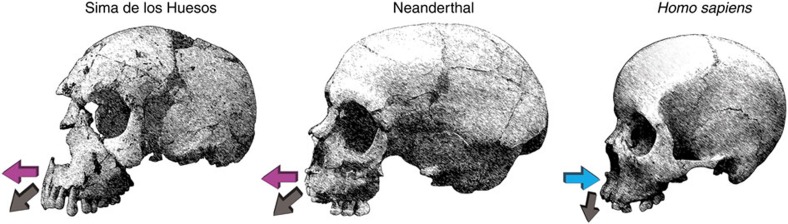
Growth directions of the maxilla. Schematic illustrates the principal growth direction of the maxilla in the Sima de los Huesos (SH) fossils, Neanderthals and modern humans. The growth remodelling identified in this study impacts growth direction in at least two ways. (i) The extensive bone deposits over the maxilla are consistent with a strong forward growth component in the fossils (purple horizontal arrows); whereas resorption over this region in the modern human face moderates forward displacement (blue horizontal arrow). (ii) Widespread deposition in the fossils combined with larger developing nasal cavities displaces prosthion downward and more anteriorly than in modern humans as indicated by the angles of the downward arrows. In humans, forward displacement is more limited with resorption compensating the anterior cortical remodelling drift of prosthion as indicated by a more downward pointing arrow. As a result of a more anterior location of prosthion in the fossil taxa, the tooth row *en bloc* drifts forward with respect to the maxillary tuberosity, thus generating the retromolar space characteristic of Neanderthals and also in some SH fossils.

The finding of similar facial bone growth remodelling activity states in the growing and developing faces of Neanderthals and SH hominins has broader implications. The SH sample and Neanderthals share a constellation of derived midfacial, dental, mandibular and glenoid cavity features that participate in a functional masticatory complex^21^. Both groups also share large floors of the nasal cavity and large palatal roofs. To generate the expanded nasal cavity in the SH hominins and Neanderthals, the nasal capsule is vertically expanded and an increased rate of remodelling of the nasal and oral components of the palate (greater resorption on the nasal floor and increased deposition on the oral lamina of the palate) likely increases downward and forward drift of this structure, resulting in larger nasopharyngeal airways earlier in development relative to *H. sapiens*. The forwardly placed mid face and nasal aperture coupled with an antero-inferior growth vector of the face may well have resulted in relatively more anterior positioning of prosthion and the tooth row *en bloc* with respect to the maxillary tuberosity, thus also generating the retromolar space characteristic of Neanderthals (Fig. 2).

Although the maxillary growth remodelling activity state is mainly depository in Neanderthals and the SH hominins, some resorption not observed in the former was noted in discrete regions of the SH fossils. This difference comprises a narrow vertical strip of resorptive activity in the SH faces where the anterior aspect of the naso-alveolar clivus meets the lateral aspect of the maxilla, slightly posterior to the canine. This could be related to the fact that there is a transverse maxillary concavity (or flexion) in this sample, whereas in Neanderthals the facial sides are flat and more parasagitally oriented. Some resorption was also observed in the SH sample in areas immediately below the nasal cavity. This is likely related to the fact that in the SH hominins (as in the African Middle Pleistocene fossils Bodo and Broken Hill) the lateral nasal crest fades out in the naso-alveolar clivus, without reaching the nasal spine to form a nasal sill, as in all Neanderthals (and some modern humans). Although the subtle remodelling differences between the fossil groups studied here could be associated with age differences in the samples, we think that this is unlikely given that their adult facial anatomy is so similar^19^,^20^,^21^,^22^,^23^. It may be that these differences represent a local growth feature of the SH hominins within an overarching facial growth plan dominated by bone deposition.

The Middle Pleistocene hominins studied here provide important information regarding the evolution of facial bone growth. The extent to which their anterior maxillary bone growth is shared with chronologically older hominins is an important consideration in assessing the appearance of evolutionary novelties in human facial development. Bone growth remodelling activity and how it has changed during the evolution of the early hominin face has been detailed in several studies of Plio-Pliocene African hominins^2^,^3^,^4^,^5^. *Au. afarensis, Au. africanus* and early *Homo* show remarkable consistency whereby all known specimens within these groups display extensive bone deposition over the maxillae^2^,^3^,^4^,^5^, something they share with living apes. Like the Middle Pleistocene hominins, *Australopithecus* and the African members of early *Homo* (as well as living apes) manifest relatively prognathic faces. An exception within *Australopithecus* is the newly discovered *Au. sediba*, which possessing a mesognathic face^28^ shows a modification in the distribution of remodelling fields relative to other members of the genus^29^. Regardless, the European Middle Pleistocene hominins display depository anterior maxillary bone growth remodelling as found in the African Pliocene and early Pleistocene hominins. Thus we suggest that bone deposition is a plesiomorphic trait among these groups, whereas the facial morphogenesis of *H. sapiens* represents a derived trait. When the characteristic bone resorptive fields of the *H. sapiens* face first appeared is difficult to ascertain given the present dearth of juvenile faces in the Lower-Middle Pleistocene record outside Sima de los Huesos. However, on limited evidence^3^, one *H. antecessor* specimen ATD6–69 (∼850 ky) does show resorptive remodelling in parts of the maxillary clivus reminiscent of that in modern humans^3^. This remodelling in ATD6–69 differs from all *Australopithecus* and all African early *Homo* specimens studied to date. Remarkably, this developmental similarity is accompanied by shared anatomical features between ATD6–69 and modern humans^30^. It may represent an evolutionary novelty only shared by the type specimen of *H. antecessor* and modern humans, but additional specimens of *H. antecessor* and other hominins of similar geological age are required to clarify this.

Changes in the onset and distribution of bone growth remodelling fields have been aptly invoked to interpret differences between Neanderthal and modern human faces, although no remodelling data were available^10^,^15^. More recently, on the basis of bone surface remodelling of fragmentary Neanderthal mandibular remains of adults, differences from modern humans were noted^27^. Here we provide definitive facial growth remodelling data from sub-adults to address the question of whether the growth of the maxilla differed in significant ways between Neanderthals, their putative ancestors from SH and modern humans. Our data on the microscopic analysis of the faces of the Devil's Tower and La Quina 18 Neanderthals reveal that anterior maxillary bone deposition is the dominant activity state in Neanderthals in contrast to the dominant resorption of modern humans. Both the SH hominin and Neanderthal facial plans are associated with extensive fields of anterior maxillary bone deposition. The ancestral pattern of anterior maxillary bone deposition observed throughout the Pliocene was not modified in these hominins. In contrast, the modern human face features resorption over the anterior maxilla associated with facial retraction. Our data contribute to the ongoing debate concerning differences in growth and development among Neanderthals and modern humans and provide new evidence that they differed at a fundamental level with regard to the activity states underlying maxillary morphogenesis. Given our current understanding of the role of bone growth remodelling in shaping facial morphogenesis and facial ontogeny, the finding of distinct processes in the faces of the SH-Neanderthal group relative to modern humans leaves no doubt that they differ in postnatal facial ontogeny in much more than just the rate of shape change.

## Methods

### Samples studied

The fossil specimens analysed in this study are shown in Table 1. The Devil's Tower Neanderthal child is housed at the Natural History Museum London. This specimen had developed the M^1^ crown and showed minimal root development but the M^2^ crown had not fully formed^31^. Incremental markings on the maxillary incisor crowns revealed an approximate age at death of 4.6 years^17^. The juvenile Neanderthal La Quina 18 (age 6–9 based on human dental standards)^13^,^18^ housed at Musée d'Archéologie nationale et Domaine National de Saint-Germain-en-Laye was only studied using portable confocal microscopy as replicas were not permitted. Following extensive cleaning of consolidant under a magnifying glass, we were able to make replicas of the Neanderthal juvenile Pech de l'Azé and the adult La Ferrassie 1 housed at the Musée de l'Homme, Paris. However subsequent analysis of the replicas in the SEM revealed that the consolidant found over most of the facial skeletons of these two specimens from the Musée de l'Homme could not be removed, preventing the identification of bone surface microanatomy. The SH sample consists of: AT-5899, a maxilla, part of Cranium 16; AT-1100; Cranium 9 and Cranium 6. All specimens are sub-adults at a biological age characterized by eruption of the second permanent molar (M^2^) and an unerupted third permanent maxillary molar (M^3^) and were aged based on the human dental standards. Cranium 6 has been ascribed to a male, based on dental characteristics, and its age at death has been estimated to be around 14 years old^19^,^20^,^21^. Cranium 9 possesses a newly erupted M^2^ and is likely to be the youngest individual in the SH sample studied. It is estimated that this individual was about 12 years of age. AT-1100 is a partial maxilla with the third molar in the process of eruption. Its estimated age at death is around 16–18 years old. AT-5899 is a well-preserved right maxilla with M^2^ erupted but without root closure. The M^3^ of this specimen has a formed crown but has not yet erupted, so it is considered to be a late adolescent. This study thus represents a cross-sectional analysis of the facial ontogeny in the SH hominins from ∼12–18 years of age (using modern developmental standards). Specimens were carefully prepared for analysis by the curators of the Institute Carlos III in Madrid, the repository of the SH fossils, removing any consolidants previously applied to the bone surfaces. The left side of Cranium 9 is largely complete so we focused our analysis on this side, providing excellent detail. AT-1100 and AT-5899 were both studied using only the SEM and showed a remarkable preservation of bone surfaces. Cranium 6 had been treated with a different consolidant than the remaining sample. It proved very difficult to remove, and so this specimen, examined with the PCSOM, provided limited remodelling information.

The modern human specimen shown in Fig. 1e derives from the collections described in ref. 4. The age at death of this specimen was 5.3 years old and had only the deciduous dentition erupted. The modern human shown in Fig. 1f derives from Archaeological collections housed at the University of Burgos. No age was known but its dental developmental status is similar to that of the SH hominins with M^2^ erupted but not the M^3^.

### Confocal and electron microscopy

High-resolution replicas of the facial skeleton of the Devil's Tower Neanderthal child were made by one of us (P.O.) using Exaflex impression material (GC America, Chicago, IL, USA). Positive replicas were prepared using Devcon 5-minute Epoxy (ITW Devcon, Danvers, MA, USA). Spur-coated positive replicas were examined by an EVO 50 SEM (Carl Zeiss, Thornwood, NY, USA) in variable pressure secondary electron emission mode (15 kV accelerating voltage, 200 pA current, 9–12 mm working distance, 100 Pa pressure). For La Quina 18, we used a portable confocal scanning optical microscope (PCSOM) as described elsewhere^32^. In cases where one side was better preserved than the other, the obtained information was mirrored on both sides. For the SH sample two microscopes were used in this study: PCSOM, and an environmental SEM. The SEM was housed at the Laboratory for Human Evolution at the University of Burgos whereas the PCSOM generally housed at NYU was brought to Spain for onsite use. Specimens were studied first hand by direct imaging of bone surfaces and of the microanatomy of bone immediately below the surface to a depth of ca. 50 μm. Field widths provided by the PCSOM ranged from ca. 500 μm (5 × lens) to 230 μm (10 × lens).

## Additional information

**How to cite this article:** Lacruz, R. S. *et al*. Ontogeny of the maxilla in Neanderthals and their ancestors. *Nat. Commun.* 6:8996 doi: 10.1038/ncomms9996 (2015).

## Supplementary Material

Supplementary Information Supplementary Figures 1-4

## Figures and Tables

**Table 1:** Specimens analysed.

Group	Specimen	Estimated age at death
Neanderthals	Gibraltar 2	Around 5 years^17^
	La Quina 18	6–9 or 7–8 (refs 13, 18)
	Pech de l'Azé	<3 years^13^
	La Ferrasie 1	Adult
SH hominins	Cranium 6	∼14 years^19^,^20^,^21^
	Cranium 9	∼12 years^19^,^20^,^21^
	Cranium 16	Late adolescent^19^,^20^,^21^
	AT-1100	16–18 years^19^,^20^,^21^

SH, Sima de los Huesos.

For estimates of age at death see the references indicated.

## References

[b1] Enlow D. H. & Hans M. G. (eds) Essentials of Facial Growth W.B. Saunders (1996).

[b2] Bromage T. G. & Boyde A. in Essentials of Facial Growth eds Enlow D. H., Hans M. G. 319–344W.B. Saunders (2008).

[b3] Lacruz R. S. . Facial morphogenesis of the earliest Europeans. PLoS ONE 8, e65199 (2013). 23762314 10.1371/journal.pone.0065199 PMC3675139

[b4] Bromage T. G. Ontogeny of the early hominid face. J. Hum. Evol. 18, 751–773 (1989).

[b5] McCollum M. A. Nasomaxillary remodeling and facial form in robust *Australopithecus*: a reassessment. J. Hum. Evol. 54, 2–14 (2008). 17825877 10.1016/j.jhevol.2007.05.013

[b6] Rosas M. & Martinez-Maza C. Bone remodeling of the *Homo heidelbergensis* mandibles, the Atapuerca-SH sample. J. Hum. Evol. 58, 127–137 (2010). 20042223 10.1016/j.jhevol.2009.10.002

[b7] Kurihara S., Enlow D. H. & Rangel R. D. Remodeling reversals in the anterior parts of the human mandible and maxilla. Angle Orthod. 50, 98–106 (1980). 6929173 10.1043/0003-3219(1980)050<0098:RRIAPO>2.0.CO;2

[b8] Lieberman D. E., McBratney B. & Krovitz G. E. The evolution and development of cranial form in *Homo sapiens*. Proc. Natl Acad. Sci. USA 300, 1134–1139 (2002). 11805284 10.1073/pnas.022440799 PMC122156

[b9] Stringer C. Modern human origins: progress and prospects. Phil. Trans. R. Soc. Lond. B 357, 563–579 (2002). 12028792 10.1098/rstb.2001.1057 PMC1692961

[b10] Ponce de Leon M. & Zollikofer C. P. E. Neanderthal cranial ontogeny and its implications for late hominin diversity. Nature 412, 534–538 (2001). 11484052 10.1038/35087573

[b11] Mitteroecker P., Gunz P., Bernhard M., Schaefer K. & Bookstein F. L. Comparison of cranial ontogenetic trajectories among great apes and humans. J. Hum. Evol. 46, 679–698 (2004). 15183670 10.1016/j.jhevol.2004.03.006

[b12] Cobb S. N. & O'Higgins P. Hominins do not share a common postnatal facial ontogenetic shape trajectory. J. Exp. Zool. B. Mol. Dev. Evol. 302, 302–321 (2004). 15211688 10.1002/jez.b.21005

[b13] Krovitz G. E. in Patterns of Human Growth and Development in the Genus Homo (eds Thompson, J. L., Krovitz, G. E. & Nelson, A. J.) 320–342Cambridge University Press (2003).

[b14] Bastir M., O'Higgins P. & Rosas A. Facial ontogeny in Neanderthals and modern humans. Proc. Royal Soc. B 274, 1125–1132 (2007). 10.1098/rspb.2006.0448 PMC2189570 17311777

[b15] Zollikofer C. P. E. Evolution of hominin cranial ontogeny. in Progress in Brain Research Vol 195 eds Michel A., Hofman Dean Falk 273–292 (Amsterdam, The Netherlands, (2012). 22230632 10.1016/B978-0-444-53860-4.00013-1

[b16] Strand Viðarsdóttir U., O'Higgins P. & Stringer C. A geometric morphometric study of regional differences in the ontogeny of the modern human facial skeleton. J. Anat. 201, 211–229 (2002). 12363273 10.1046/j.1469-7580.2002.00092.x PMC1570912

[b17] Smith, . Dental evidence for ontogenetic differences between modern humans and Neanderthals. Proc. Natl Acad. Sci. USA 107, 20923–20928 (2010). 21078988 10.1073/pnas.1010906107 PMC3000267

[b18] Minugh-Purvis N. in *Human Evolution through Developmental Change* (eds Minugh-Purvis N., McNamara K. J. 479–498Johns Hopkins University Press (2002).

[b19] Arsuaga J. L., Martinez I., Gracia A. & Lorenzo C. The Sima de los Huesos (Sierra de Atapuerca, Spain). A comparative study. J. Hum. Evol. 33, 219–281 (1997). 9300343 10.1006/jhev.1997.0133

[b20] Arsuaga J. L., Martinez I., Gracia A., Carretero J. M. & Carbonell E. Three new human skulls from the Sima de los Huesos Middle Pleistocene site in Sierra de Atapuerca, Spain. Nature 362, 534–537 (1994). 8464493 10.1038/362534a0

[b21] Arsuaga J. L. . Neanderthal roots: cranial and chronological evidence from Sima de los Huesos. Science 344, 1358–1363 (2014). 24948730 10.1126/science.1253958

[b22] Stringer C. The status of *Homo heidelbergensis* (Schoetensack 1908). Evol. Anthropol. 21, 101–107 (2012). 22718477 10.1002/evan.21311

[b23] Rightmire G. P. Deep roots for Neanderthals. Nature 389, 917–918 (1997). 9353114 10.1038/40024

[b24] Meyer M. . Nuclear DNA sequences from the hominin remains of Sima de los Huesos, Atapuerca, Spain. In *Proceedings of the European Society for the study of Human Evolution (ESHE) 4* (5th Annual Meeting of the ESHE, (2015).

[b25] Martinez-Maza C., Rosas A. & Nieto-Díaz M. Postnatal changes in the growth dynamics of the human face revealed from bone modelling patterns. J. Anat. 223, 228–241 (2013). 23819603 10.1111/joa.12075 PMC3972044

[b26] Enlow D. H. & Bang S. Growth and remodeling of the human maxilla. Am. J. Orthod. 51, 446–464 (1965). 14287831 10.1016/0002-9416(65)90242-3

[b27] Martinez-Maza C., Rosas A., Garcia-Vargas S., Estalrrich A. & de la Rasilla M. Bone remodeling in Neanderthal mandibles from the El Sidron site (Asturias, Spain). Biol. Lett. 7, 593–596 (2011). 21307043 10.1098/rsbl.2010.1188 PMC3130222

[b28] Berger L. R. . *Australopithecus sediba*: a new species of *Homo*-like australopith from South Africa. Science 328, 195–204 (2010). 20378811 10.1126/science.1184944

[b29] Lacruz R. S. . Distinct growth of the nasomaxillary complex in *Au. Sediba*. Sci. Rep. 5, 15175 (2015). 26469387 10.1038/srep15175 PMC4606807

[b30] Bermudez de Castro J. M. . A hominid from the Lower Pleistocene of Atapuerca, Spain: Possible Ancestor to Neanderthals and modern humans. Science 276, 1392–1395 (1997). 9162001 10.1126/science.276.5317.1392

[b31] Dean M. C., Stringer C. B. & Bromage T. G. Age at death of the Neanderthal child from Devil's Tower, Gibraltar and the implications for studies of general growth and development in Neanderthals. Am. J. Phys. Anthropol. 70, 301–309 (1986). 3752228 10.1002/ajpa.1330700305

[b32] Bromage T. G. . Confocal scanning optical microscopy of a 3-million year old *Australopithecus afarensis* femur. Scanning 31, 1–10 (2009). 19191265 10.1002/sca.20139

