# Supplementary material for: Ontogeny of the maxilla in Neanderthals and their ancestors
Source: Nat Commun. 2015 Dec 7;6:8996. doi: 10.1038/ncomms9996 (PMC4686851; doi:10.1038/ncomms9996)

Supplementary Figure 1. Electron micrographs of Devil's Tower (Gibraltar 2) Neanderthal. All images are taken on the right side of the maxilla of DT Neanderthal except (D) which represent perikymata taken on the central incisor to show the pristine preservation of bone and enamel microanatomy. **A-C** images all represent bone deposition. Perikymata were imaged to asses preservation in all aspects of the replica.

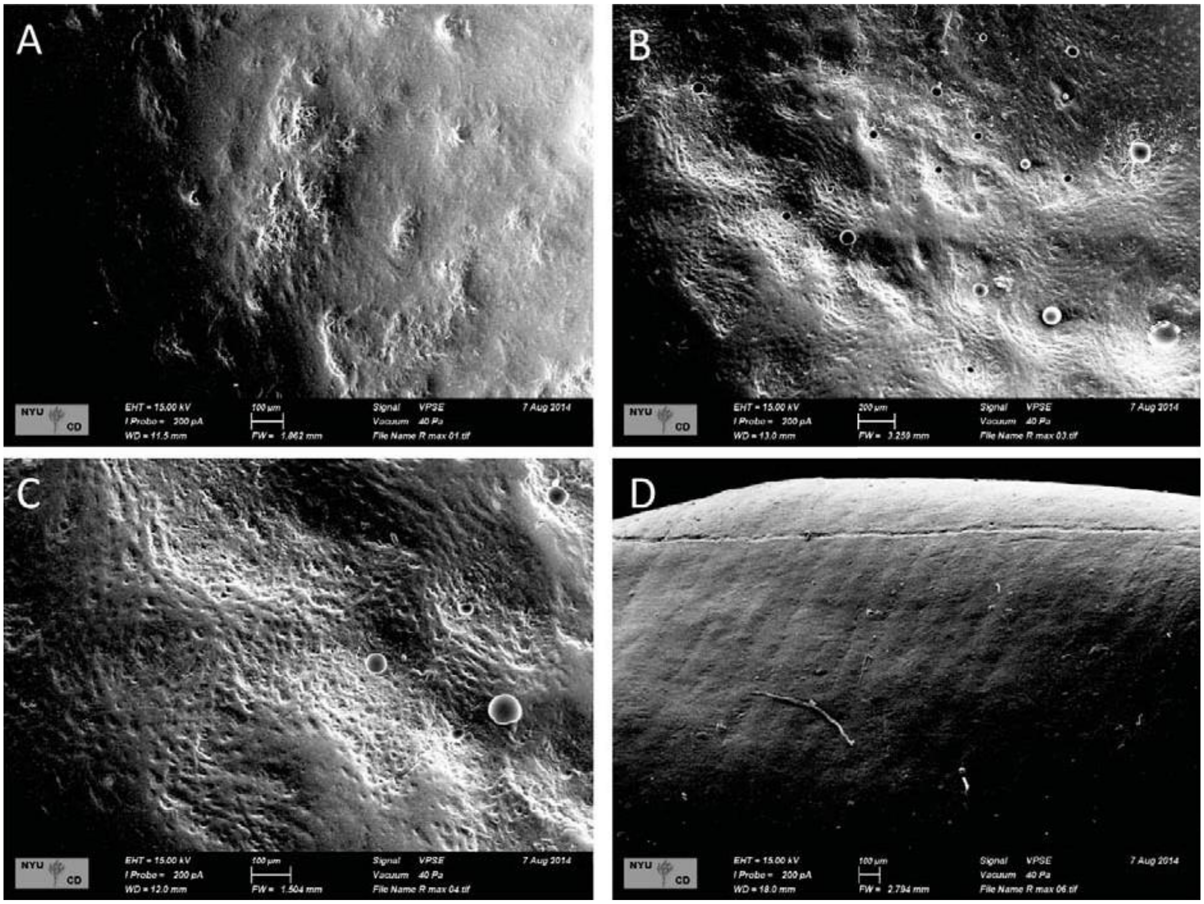

Supplementary Figure 2. Remodeling in La Quina 18 Neanderthal. This image shows surface remodeling in the clivus of La Quina 18 Neanderthal imaged with the PCSOM. Only bone deposition can be identified in this stereopair. Field width: ~500 μm.

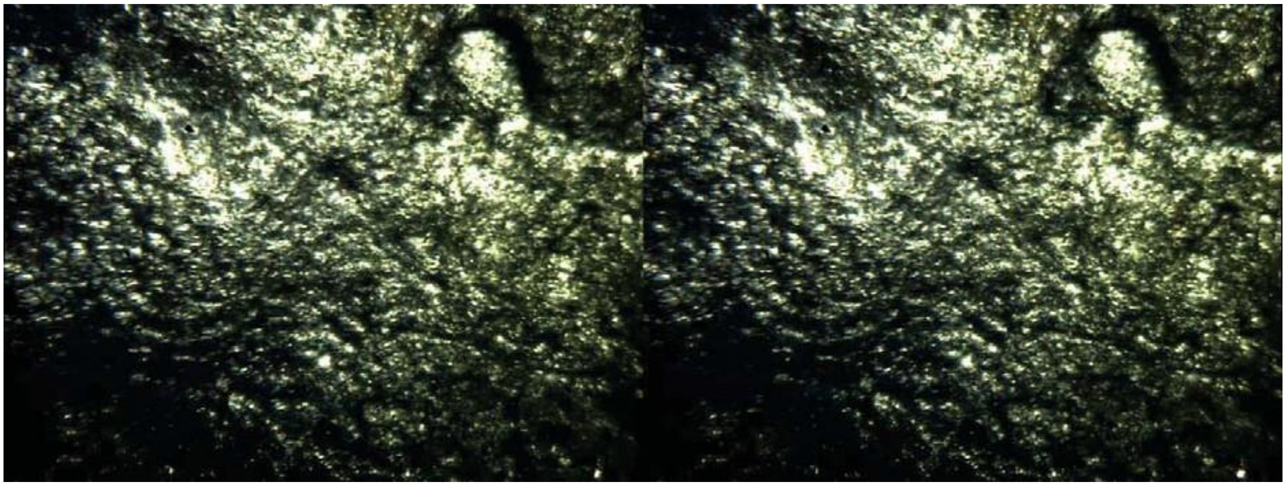

Supplementary Figure 3. Remodeling in SH hominins. Electron micrographs taken directly on the fossils using an environmental SEM. **(A)** Right aspect of the nasal aperture of the maxilla of AT-1100 showing resorption. **(B)** AT- 5899 alveolar bone also showing the root of the first premolar. The alveolar bone shows resorption. **(C)** Resorption on the lateral maxilla of AT-5899 taken over the narrow strip of resorption on the lateral aspect of the maxilla described in the text and found in all specimens from SH studied **(D)** Resorption islet on the alveolar bone of the canine in AT-5899. **(E)** Deposition on the maxilla lateral to the nasal aperture in AT-5899.

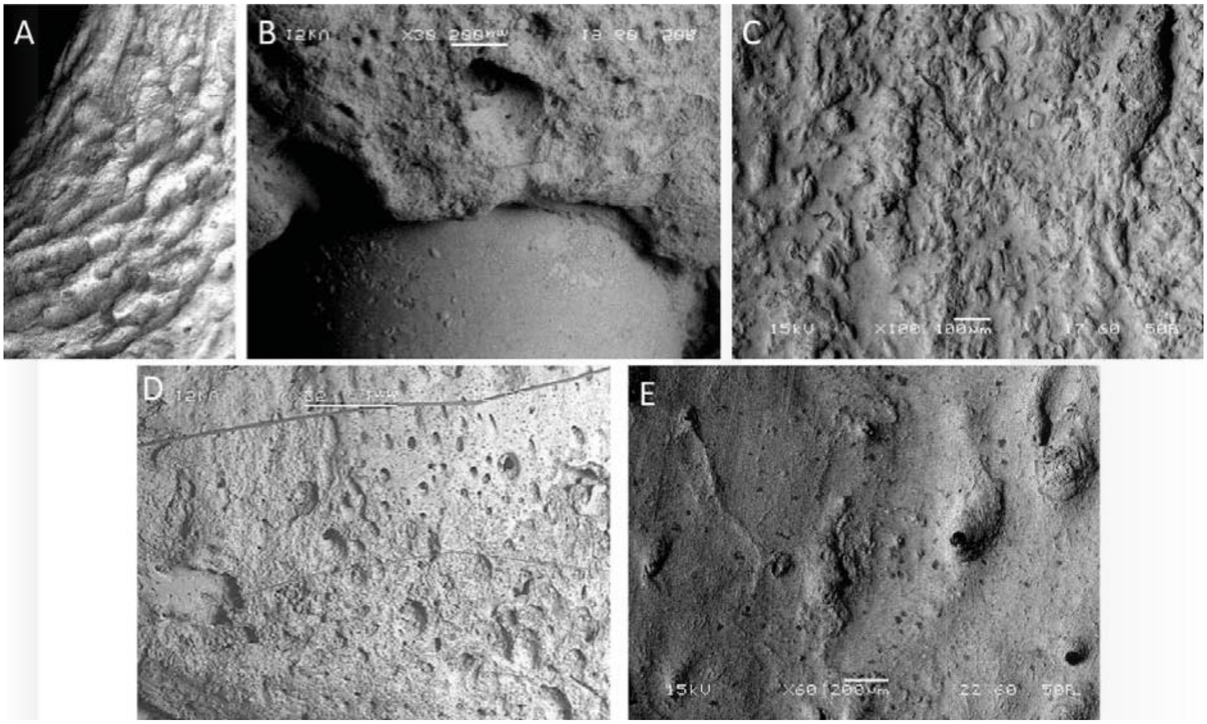

Supplementary Figure 4: Hominin facial maps: Bone resorption and bone deposition are shown in the faces of La Quina 18 and the SH hominins. **(A)** La Quina 18 Neanderthal, **(B)** SH hominin. Cranium 9**, (C)** SH hominin. Cranium 6, **(D)** SH hominin. Maxilla AT-5899 part of Cranium 16 frontal view, **(E)** SH hominin. Maxilla AT-5899 part of Cranium 16 lateral view. **(F)** SH hominin. Isolated maxilla AT-1100. **A-C:** Imaged using portable confocal microscopy. The circles represent the areas investigated and from which we obtained remodeling data. We have not mirrored the information from left and right. Each spot represents our raw data. **D-F**: Specimens imaged using electron microscopy. The facial map of Devil's Tower Neanderthal is shown in Figure 1.Deposition is represented as purple colour. Resorption is represented as blue. Areas not sampled or for which remodeling data could not be obtained are shown in ivory colour.

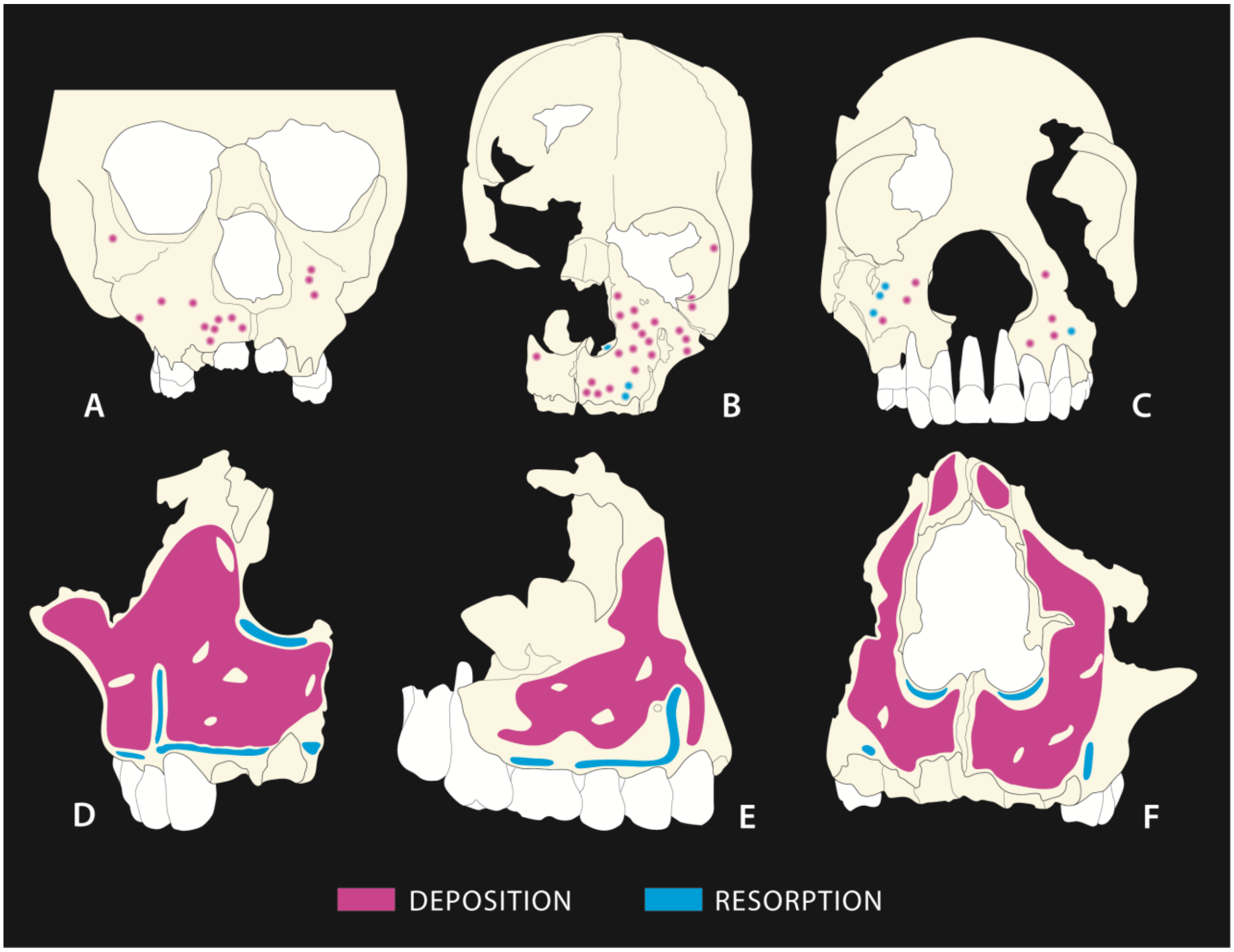

Supplement: Supplementary Information — Supplementary Figures 1-4 [file ncomms9996-s1.pdf]
